# Diverse Forage Enhances the Antimicrobial Potency of Australian Honey

**DOI:** 10.1002/mbo3.70238

**Published:** 2026-02-23

**Authors:** Kenya E. Fernandes, Andrew Dong, Jamie Ayton, Leanne Groves, Kerrie Graham, Peter Brooks, Nural Cokcetin, Dee A. Carter

**Affiliations:** ^1^ School of Life and Environmental Sciences University of Sydney Sydney New South Wales Australia; ^2^ Sydney Institute for Infectious Diseases University of Sydney Sydney New South Wales Australia; ^3^ NSW Department of Primary Industries and Regional Development, Wagga Wagga New South Wales Australia; ^4^ School of Science, Technology and Engineering University of the Sunshine Coast Maroochydore Queensland Australia

**Keywords:** antimicrobial activity, Apis mellifera, Australian honey, natural products

## Abstract

Antimicrobial resistance is a critical global health crisis, driving renewed interest in natural alternatives like honey. Australia's diverse native flora offers unique opportunities for producing bioactive honeys, yet their antimicrobial potential remains underexplored. This study aimed to systematically evaluate the antimicrobial activity and chemical composition of Australian honeys from post‐bushfire New South Wales landscapes and to identify key drivers of bioactivity and their therapeutic potential. Antimicrobial activity was assessed against *Staphylococcus aureus* and *Escherichia coli* using broth microdilution methods, measuring both total activity and non‐peroxide activity. Comprehensive chemical profiling included bioactive compounds, quality parameters, sugars, organic acids, amino acids, volatiles, and secondary metabolites, assessed using standardized methodologies and ¹H‐Nuclear Magnetic Resonance spectroscopy. Statistical analyses included correlation analysis, LASSO regression modeling, and principal component analysis. Most honeys exhibited strong antimicrobial activity, with 77% achieving MICs of ≤ 10% (w/w) against both pathogens, and 25% achieving ≤ 5% (w/w) against at least one pathogen. Honeys produced from mixed flora had consistently high levels of antibacterial activity, while monofloral honeys were much more variable. H_2_O_2_ was the strongest single factor associated with antibacterial potency, explaining 45%–46% of the variability in MIC values among samples. Including additional chemical parameters in multivariate models improved the ability to predict antibacterial strength, explaining up to 59% of the variation for *S. aureus*, and 73% for *E. coli*. The superior performance of mixed‐flora samples challenges conventional assumptions favoring monofloral products and supports biodiversity‐focused beekeeping practices, providing a foundation for developing Australian honeys as therapeutic alternatives while supporting sustainable industry recovery.

## Graphical abstract

Australian native flora honeys show strong, multicomponent antimicrobial activity at therapeutically relevant levels. Mixed‐flora honeys consistently outperform monofloral varieties, with diverse nectar sources supporting bee health and driving multifactor chemical complexity in honey.

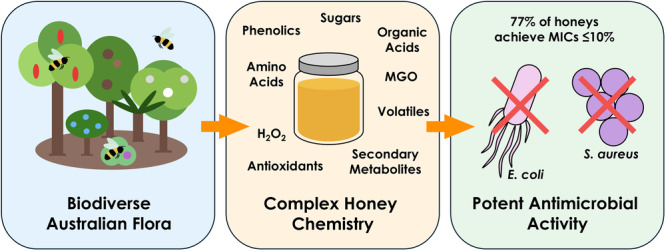

## 1 Introduction

Antimicrobial resistance is a critical global health crisis, threatening human, animal, and ecosystem health (Ho et al. 2025). Infections caused by drug‐resistant microbes are increasingly difficult and costly to treat, contributing substantially to global morbidity and mortality (Murray et al. 2022). In response, there is renewed interest in alternative antimicrobials, particularly natural products. Honey has re‐emerged as a promising agent due to its broad‐spectrum antimicrobial properties and long history of use in wound care (Ogwu and Izah 2025; Clark and Adcock 2018). Honey‐based treatments have demonstrated efficacy against drug‐resistant pathogens in vitro, and bacterial resistance is considered unlikely due to multiple antimicrobial mechanisms (Blair et al. 2009).

The antimicrobial potency of honey is influenced not only by its floral source but also by bee‐mediated processing during honey production, suggesting that the interaction between nectar chemistry and bee physiology is a key determinant of bioactivity. In this context, Australia's native flora provides an exceptional but underexplored source of chemically distinctive nectar, which could give rise to honeys with unique bioactive properties. With the world's richest assemblage of *Eucalyptus* and *Leptospermum* species, alongside nectar sources not found elsewhere, Australian landscapes yield honeys with distinctive chemical profiles (Ayton et al. 2025; Roshan et al. 2017). Surveys have shown that many possess antibacterial activity at therapeutically useful levels, with honeys from native plants like marri *(Corymbia calophylla)*, jarrah *(Eucalyptus marginata)*, and jellybush *(Leptospermum polygalifolium)* displaying particularly strong effects (Irish et al. 2011; Fernandes et al. 2024a). The availability and diversity of nectar sources also sustain bee health and colony function, which in turn influences the composition, and bioactivity of honey produced (Fernandes et al. 2023). The Australian honey industry is closely tied to this ecological richness: an estimated 70% of commercial honey is derived from native forests and bushland, contributing to an annual production of more than 37,000 tonnes (Spicer and McGaw 2020; Clarke and Le Feuvre 2021).

In 2020, extensive bushfires across New South Wales devastated vast areas of forest, destroying hives, bees, floral resources, and critical infrastructure. These fires not only threatened the livelihoods of beekeepers but also disrupted the ecological networks that support honey production, including native plant‐pollinator interactions critical for nectar availability, floral diversity, and colony nutrition. Such disturbances highlight the vulnerability of Australia's native ecosystems and the honey industry, while also presenting a timely opportunity to evaluate honey from post‐bushfire landscapes and inform strategies for sustainable industry recovery. Combined with the region's floral richness, significant production scale, and emerging niche therapeutic markets, these circumstances underscore the value of systematic scientific investigation into Australian honeys.

Understanding what drives antimicrobial activity in Australian honeys requires examining honey's multiple, complementary mechanisms. In most honeys, the enzyme glucose oxidase, introduced by bees during honey production, generates H_2_O_2_ upon dilution, producing reactive oxygen species that damage microbial cells (Machado De‐Melo et al. 2018). High osmolarity and low pH further create inhospitable conditions for microbes (Almasaudi 2021). Beyond these general properties, honeys contain diverse bioactive compounds derived from nectar, some of which are modified through bee activity. Many honeys are rich in phytochemicals, including phenolic acids and flavonoids, which provide both antioxidant and direct antibacterial effects (Machado De‐Melo et al. 2018). A notable example is manuka honey, derived from *Leptospermum* nectar, whose activity is largely attributed to methylglyoxal, a compound that disrupts bacterial metabolism and cell structure (Blair et al. 2009). Together, these mechanisms confer honey with broad‐spectrum activity against a wide range of pathogens.

In this study, 56 honeys collected from beekeepers across New South Wales were analyzed to examine the drivers underlying their antimicrobial activity. Activity was measured against *Staphylococcus aureus* and *Escherichia coli*, assessing both total activity (TA) and non‐peroxide activity (NPA) to differentiate between peroxide‐dependent and other mechanisms. In parallel, comprehensive chemical profiling was conducted, encompassing bioactive traits, quality and compositional parameters, sugars, organic acids, amino acids, volatiles, and secondary metabolites. By integrating microbiological and chemical data, the aim was to identify honeys with potent antibacterial activity and to determine which natural constituents contribute most strongly to their bioactivity, with implications for therapeutic applications and post‐bushfire industry recovery.

## 2 Materials and Methods

### 2.1 Honey Samples and Preparation

**Figure 1:**
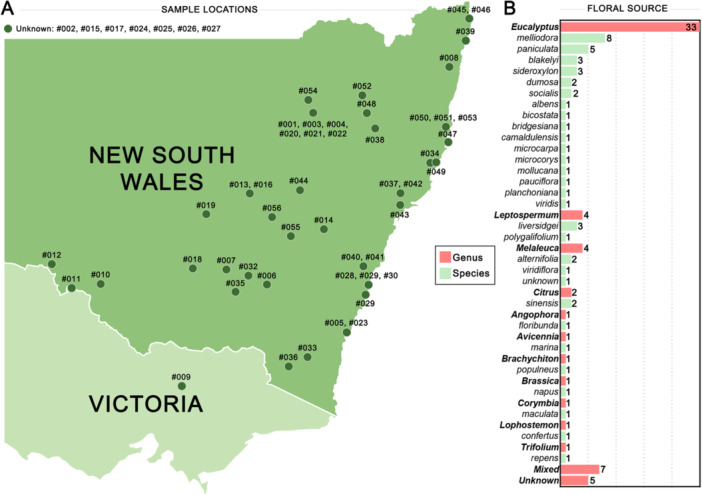
Honey sample collection details. (A) Locations of honey samples collected across New South Wales and Victoria. (B) Reported floral source of honey samples with genus (red) and species (green) shown.

A total of 56 honey samples were received from beekeepers and honey packers across New South Wales and Victoria between 2021 and 2024. The geographic distribution of sample collection sites is shown in Figure 1. Samples were securely transported to the laboratory in sealed plastic containers at ambient temperature, subsampled, and stored in amber colored glass jars at 4°C in the dark. Each sample was assigned a unique reference code, and accompanying metadata provided by beekeepers including collection date, location, and floral source, were recorded in a central database (Table S1). Floral source identification was based on beekeeper knowledge, considering the predominant flora available for nectar foraging, the geographic location of the apiary, and the sensory characteristics of the honey. Samples consisted of predominantly single‐floral *Eucalyptus* honeys (*n* = 33, representing 12 species), *Leptospermum* honeys (*n* = 4, representing three species), *Melaleuca* honeys (*n* = 4, representing two species), and other single‐floral sources (*n* = 8), along with mixed‐flora honeys (*n* = 7) (Figure 1B). Unless otherwise specified, all honey samples were mixed thoroughly with a spatula, incubated at 35°C for 15 min to dissolve sugar crystals, diluted to the target concentration in sterile water, and vortexed thoroughly before use. Unless otherwise stated in the relevant assay descriptions, measurements represent a single analytical determination per honey sample.

### 2.2 Microorganisms and Culture Conditions

One Gram‐positive bacterium (*Staphylococcus aureus* ATCC29213) and one Gram‐negative bacterium (*Escherichia coli* ATCC25923) were used for antimicrobial testing. Bacteria were maintained as glycerol stocks at—80°C, grown on Nutrient Agar (NA; Oxoid) and incubated at 30°C for 24 h before use.

### 2.3 Antimicrobial Susceptibility Testing

Honey samples were sterilized by gamma irradiation at 15 Gy (Steritech, Wetherill Park, NSW) prior to testing. Gamma irradiation is widely used for sterilization of medical and research‐grade honeys, and effectively eliminates microbial contaminants, including spores, while preserving antibacterial and physicochemical properties (Molan and Allen 1996; Faraz et al. 2023). Antimicrobial susceptibility testing by broth microdilution was then performed in accordance with CLSI guidelines for aerobic bacteria (Clinical and Laboratory Standards Institute CLSI 2012) with minor modifications. Briefly, inocula were prepared from colonies growing on agar plates to a final concentration of 2 × 10^5^–8 × 10^5^ CFU/mL by adjusting to an absorbance of between 0.08 and 0.1 at 540 nm. Assays used Mueller‐Hinton Broth (MHB; Oxoid) supplemented with 20 mg Ca^++^/L and 10 mg Mg^++^/L. Honeys were assayed at 30, 25, 20, 15, 10, and 5% (w/w) diluted in either sterile water (for total activity) or freshly prepared 5600 U/mL catalase solution (for non‐peroxide activity). To maintain proper MHB concentration, bacterial inoculum was prepared in 2 × MHB and combined 1:1 with honey solutions, resulting in a final 1 × MHB concentration in each well. Assays were performed in flat‐bottom 96‐well microplates and incubated without agitation at 35°C for 18 h. The MIC was determined visually and defined as the lowest honey concentration at which growth was completely inhibited with no visible turbidity. Three independent repeats were performed for each honey sample.

### 2.4 Hydrogen Peroxide (H_2_O_2_) Assays

H_2_O_2_ production was measured in honey samples using peroxide test strips (Precision Laboratories Cat. No. IS124‐50S). Honey samples were diluted to 25% (w/w) in sterile water and incubated at room temperature for 2 h. This incubation period was selected based on previous work (Fernandes et al. 2024a), which demonstrated that H_2_O_2_ concentration at 2 h strongly correlates with both peak H_2_O_2_ concentration and total H_2_O_2_ produced across diverse honey samples. Following incubation, H_2_O_2_ test strips were then immersed in honey samples for 1 s and the excess shaken off. Test strips were allowed to develop for 10 s before a scanner was used to capture an image. The image was converted to grayscale (image > type > 16 bit) in ImageJ (version 1.52a National Institutes of Health, USA), and the grayscale intensity values from 0 to 255 were measured (analyze > set measurements > mean gray value). Known concentrations of H_2_O_2_ were used to generate a standard curve to convert the grayscale color intensity data back to parts per million (ppm).

### 2.5 Phenolics, Antioxidants, and Color Assays

The Fast Blue BB (FBBB) assay for measuring phenolic content and the ferric‐reducing antioxidant power (FRAP) assay for measuring antioxidant content were performed as previously described in (Fernandes et al. 2024a). Each assay was conducted using three independent technical replicates per honey sample. For color intensity, honey samples were diluted to 50% (w/w) in MilliQ water, and absorbance at 450 and 720 nm was measured using a UV/Vis spectrophotometer (Specord S600) in disposable plastic cuvettes with 10 mm optical pathlength. Color intensity was then calculated using the equation ${(A}_{450}-A_{720})\times 1000$ and expressed as mAU. Measurements were performed in triplicate for each honey sample.

### 2.6 Methylglyoxal and Dihydroxyacetone Assays

Honey samples were derivatised with O‐(2,3,4,5,6‐pentafluorobenzyl)hydroxylamine HCl and analyzed against Anisole by HPLC with 263 nm detection as outlined in (Pappalardo et al. 2016).

### 2.7 Quality and Composition Analysis

Physicochemical analyses were conducted following standard methodologies established by the International Honey Commission (IHC) (Bogdanov et al. 1997) and as outlined in (Ayton et al. 2025). Water content was determined using a refractometric method following IHC Method 1. Water insoluble solids were measured following IHC Method 8. Electrical conductivity was assessed following IHC Method 2. Free acidity and pH were measured using a benchtop pH meter (SmartChem‐Lab) following IHC Method 4. Hydroxymethylfurfural content was quantified by high‐performance liquid‐chromatography (HPLC) following IHC Method 5.1 and guidelines in IHC Method 5.2. Diastase activity was assessed using the Phadebas assay following IHC Method 6.2. Fructose, glucose, and sucrose were quantified by HPLC following IHC Method 7.2.

### 2.8 Nuclear Magnetic Resonance‐Based Profiling

NMR‐based profiling included quantification of sugars, acids, amino acids, fermentation markers, and secondary metabolites. Honey samples were submitted to Bruker BioSpin GmhH & Co. KG (Ettlingen, Germany) for ^1^H‐Nuclear Magnetic Resonance (NMR)‐based profiling. Analyses were performed using the Honey Profiling Platform V3.1.4 under DIN EN ISO/IEC 17025:2018 accreditation (certificate D‐PL‐22753‐01‐00), following internal method AA‐72‐03‐19. Sample preparation and acquisition were conducted according to Bruker standard operating procedures.

### 2.9 Statistical Analysis

The Shapiro‐Wilk normality test was used to test data for normal distribution. For statistical analysis, MIC readings above the tested maximum of 30% (w/v) honey were assigned a value of 35%. For all correlations between antimicrobial activity and the physical and chemical properties of honey, MIC values were subtracted from 35 to reverse their direction so that variables related with increased antimicrobial activity would appear as positively rather than negatively correlated. Associations between variables used Spearman's rank correlations, which determines the strength and direction of monotonic relationships but does not assume linearity, or linear regressions which do assume linearity. A correlation matrix was constructed between antimicrobial outcomes and chemical variables. To enhance interpretability, correlation plots were visualized with a fixed scale (–1 to 1), and statistically significant associations were highlighted. To identify the most informative predictors of antimicrobial activity, least absolute shrinkage and selection operator (LASSO) regression was conducted. Variables with non‐zero coefficients in the regularized model were interpreted as key contributors to antimicrobial efficacy. Final multiple linear regression models incorporating these selected variables were fitted and visualized to assess model performance. Principal component analysis (PCA) was performed to visualize the multivariate structure of the dataset. *p*‐values < 0.05 were considered significant. Data were analyzed using Prism 5 (GraphPad Inc), and R 4.2.2 (R Core Team).

## 3 Results

A total of 56 honeys sourced from beekeepers across New South Wales were surveyed to assess their antimicrobial activity and chemical composition (with collection sites shown in Figure 1A and full sample details in Table S1). Floral sources were determined by beekeeper assessment and were predominantly native Australian species, with *Eucalyptus* species being the most common nectar source, followed by *Leptospermum* and *Melaleuca*. Other native taxa represented include *Angophora*, *Avicennia*, *Brachychiton*, *Corymbia*, and *Lophostemon* (Figure 1B). A smaller number of samples contained nectar from exotic plants such as *Citrus, Brassica*, and *Trifolium*. Seven samples were classified as mixed floral, while five had an unknown floral origin.

### 3.1 Antimicrobial Activity Was Widespread and Highly Variable Across Honey Samples

**Figure 2:**
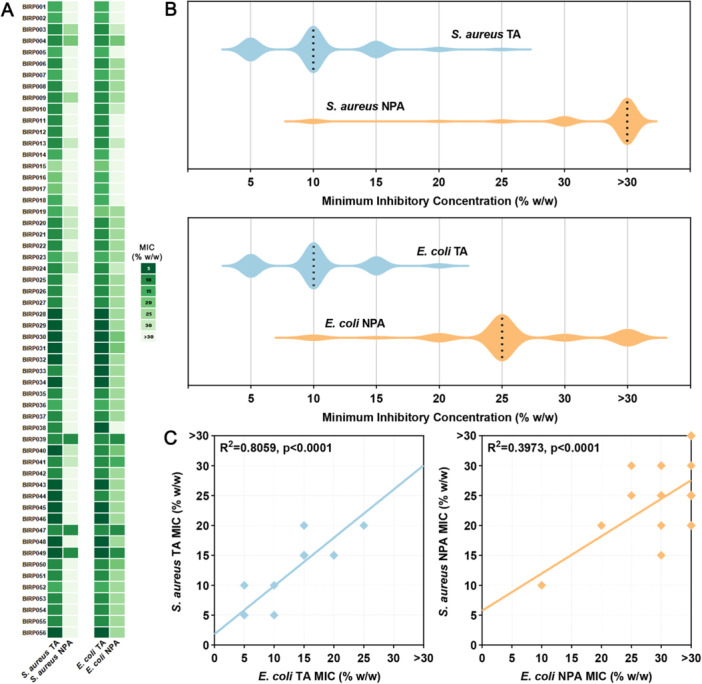
Antimicrobial activity of honey samples measured by broth microdilution assay. (A) Heatmap displaying the minimum inhibitory concentration (MIC) for each honey sample, with *S. aureus* on the left and *E. coli* on the right. TA = total activity; NPA = non‐peroxide activity. The % (w/w) refers to the concentration of honey diluted in sterile water (for TA) or catalase solution (for NPA). (B) Violin plots showing the distribution of MIC values for *S. aureus* (top) and *E. coli* (bottom). (C) Correlations between *S. aureus* and *E. coli* TA (left) and NPA (right) for all honey samples.

The majority of honey samples demonstrated strong antimicrobial activity against both bacterial pathogens. For total activity, most samples inhibited *S. aureus* at concentrations of 10% (w/w) or lower, with MIC values ranging from 5% to 25% (w/w) (median: 10%) (Table 1; Figure 2A,B). *E. coli* showed a similar pattern, with MIC values ranging from 5% to 20% (w/w) (median: 10%). Neutralization of H_2_O_2_ using 5600 U/mL catalase solution to assess non‐peroxide activity resulted in a substantial reduction in potency across most samples. For *S. aureus*, non‐peroxide MIC values ranged from 10%–> 30% (w/w) (median: > 30%), and for *E. coli* values ranged from 10%–> 30% (w/w) (median: 25%). Despite this decrease, many samples retained substantial residual activity, particularly against *E. coli*, indicating contributions from bioactive compounds other than H_2_O_2_.

**Table 1:** Details of antimicrobial activity and chemical properties measured for honey samples in this study.

**Measurement**	**Median**	**Range**	**Standard limits**	**% Samples exceeding limits**
*Antimicrobial activity*				
*S. aureus* TA MIC (% w/w)	10	5–25	N/A	N/A
*S. aureus* NPA MIC (% w/w)	35	10–> 30	N/A	N/A
*E. coli* TA MIC (% w/w)	10	5–20	N/A	N/A
*E. coli* NPA MIC (% w/w)	25	10–35	N/A	N/A
*Bioactive properties*				
Hydrogen peroxide at 2 h (ppm)	13	0–45	N/A	N/A
Phenolic content (mg GAE/kg)	99	6–360	N/A	N/A
Antioxidant activity (µmol Fe^2^^+^ /kg)	626	259–1275	N/A	N/A
Color intensity (mAU)	529	148–2392	N/A	N/A
Methylglyoxal (mg/kg)	0	0–341	N/A	N/A
Dihydroxyacetone (mg/kg)	0	0–1102	N/A	N/A
*Quality and composition*				
Water Content (g/100 g)	16.4	13.4–20.0	< 20	0
Water insoluble solids (g/100 g)	0.04	0.00–0.42	< 0.1	16
Electrical conductivity (mS/cm)	0.35	0.13–1.91	< 0.8	7
Free acidity (mEg/kg)	15.6	7.0–37.0	< 50	0
pH	4.1	3.75–5.21	N/A	N/A
Hydroxymethylfurfural (mg/kg)	4.2	0.0–242.6	< 40	4
Diastase activity (DN, Schade Scale)	19.6	1.9–46.6	> 8	4
*Sugars*				
Fructose (g/100 g)	39.3	33.0–53.3	N/A	N/A
Glucose (g/100 g)	29.7	10.6–40.2	N/A	N/A
Fructose + Glucose (g/100 g)	69.4	57.3–76.0	> 60	2
Sucrose (g/100 g)	0.0	0.0–8.1	< 5	4
Gentiobiose (g/100 g)	—	—	N/A	N/A
Maltose (g/100 g)	0.9	0.00–2.56	N/A	N/A
Maltotriose (g/100 g)	—	—	N/A	N/A
Mannose (g/100 g)	—	—	N/A	N/A
Melezitose (g/100 g)	—	—	N/A	N/A
Raffinose (g/100 g)	0.22	0.00–0.69	N/A	N/A
Turanose (g/100 g)	2.3	1.39–3.32	N/A	N/A
*Organic acids*				
Acetic acid (mg/kg)	18.74	0.00–90.42	N/A	N/A
Citric acid (mg/kg)	0.00	0.00–333.01	N/A	N/A
Formic acid (mg/kg)	34.98	13.20–138.61	N/A	N/A
Fumaric acid (mg/kg)	0.00	0.00–8.99	N/A	N/A
Lactic acid (mg/kg)	23.09	0.00–217.28	N/A	N/A
Malic acid (mg/kg)	0.00	0.00–501.51	N/A	N/A
Pyruvic acid (mg/kg)	20.53	0.00–40.82	N/A	N/A
Quinic acid (mg/kg)	—	—	N/A	N/A
Succinic acid (mg/kg)	9.94	0.00–104.91	N/A	N/A
*Amino acids*				
Alanine (mg/kg)	13.41	0.00–27.66	N/A	N/A
Aspartic acid (mg/kg)	—	—	N/A	N/A
Glutamine (mg/kg)	—	—	N/A	N/A
Leucine (mg/kg)	0.00	0.00–58.29	N/A	N/A
Proline (mg/kg)	516.63	198.44–948.26	N/A	N/A
Valine (mg/kg)	0.00	0.00–13.87	N/A	N/A
Tyrosine (mg/kg)	0.00	0.00–64.43	N/A	N/A
Phenylalanine (mg/kg)	0.00	0.00–279.92	N/A	N/A
*Volatiles*				
2,3‐Butanediol (mg/kg)	31.83	0.00–154.20	N/A	N/A
Acetoin (mg/kg)	27.80	0.00–167.12	N/A	N/A
Ethanol (mg/kg)	10.00	0.00–1455.61	N/A	N/A
*Secondary metabolites*				
3‐Phenyllactic acid (mg/kg)	0.00	0.00–694.54	N/A	N/A
Kynurenic acid (mg/kg)	—	—	N/A	N/A
Shikimic acid (mg/kg)	—	—	N/A	N/A

MIC distributions were broadly comparable between the two bacterial species for total activity but diverged more for non‐peroxide activity (Figure 2C). Significant positive correlations were observed between total activity values (*R* ^2^ = 0.8059, *p* < 0.0001) and between non‐peroxide activity values (*R* ^2^ = 0.3973, *p* < 0.0001) for *S. aureus* and *E. coli*, suggesting some shared mechanisms of antibacterial activity. However, the weaker correlation observed for NPA suggests greater mechanistic divergence in non‐peroxide effects.

### 3.2 Chemical Composition Varied Markedly Across Honey Samples

**Figure 3:**
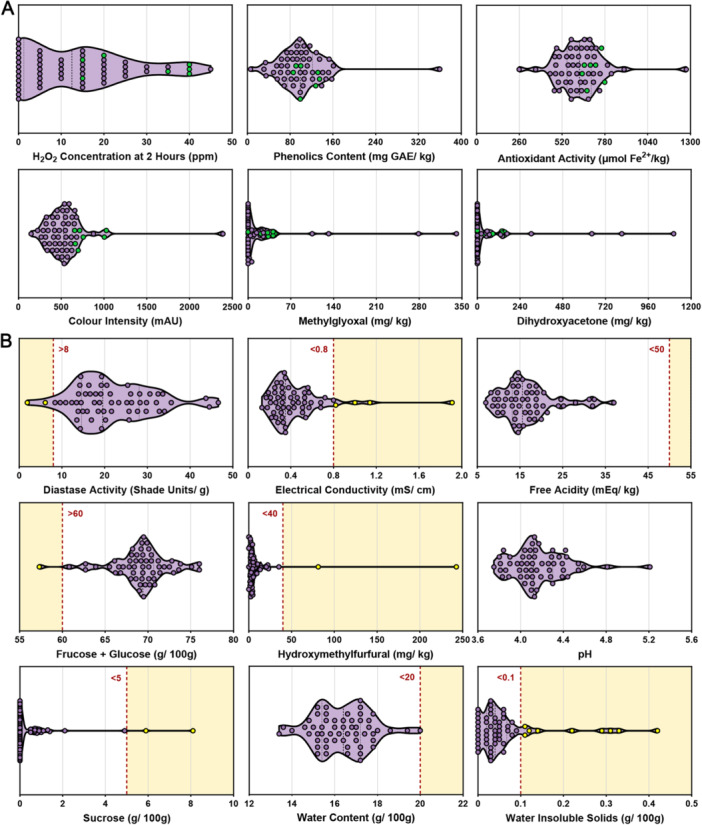
Distribution of key chemical parameters in honey samples. Violin plots showing the distribution of chemical properties associated with (A) bioactive properties, with mixed‐flora samples indicated by green dots and (B) quality and compositional parameters, with samples exceeding industry standard limits indicated by yellow dots. Red dotted lines show the corresponding industry standard thresholds.

Chemical profiling revealed substantial heterogeneity in bioactive properties across the 56 honey samples (Table 1; Figure 3A). H_2_O_2_ production at the 2 h timepoint ranged from 0 to 45 ppm (median: 13). Phenolic content, an important class of plant‐derived bioactive compounds, ranged from 6 to 360 mg GAE/kg (median: 99). Antioxidant activity and color intensity, which often correlate with phenolic content, ranged from 259 to 1275 μmol Fe²⁺/kg (median: 626) and 148–2392 mAU (median: 529), respectively. Methylglyoxal, the signature compound of *Leptospermum* honeys, was detected at significant levels in a minority of samples (range: 0–341 mg/kg, median: 0), along with its precursor dihydroxyacetone (range: 0–1102 mg/kg, median: 0).

Quality and compositional parameters were generally within accepted industry standards with a few outliers (Table 1; Figure 3B). Most honeys met all specifications, with 13 samples out of specification in one parameter, two in two parameters, and one in three parameters. Water content ranged from 13.4 to 20.0 g/100 g (median: 16.4), with all samples meeting the < 20 g/100 g standard. Free acidity ranged from 7.0 to 37.0 mEq/kg (median: 15.6), with all samples well below the standard of < 50 mEq/kg. Most samples also conformed to thresholds for water‐insoluble solids (range: 0.00–0.42 g/100 g, median: 0.04), although 16% exceeded the < 0.1 g/100 g standard. A smaller proportion exceeded thresholds for electrical conductivity (7% above < 0.08 mS/cm, range 0.13–1.91, median: 0.35), diastase activity (4% below > 8 DN, range 1.9–46.6, median: 19.6), hydroxymethylfurfural (4% above < 40 mg/kg, range: 0.0–242.6, median: 4.2), sucrose (4% above < 5 g/100 g, range: 0.0–8.1, median: 0.0), and combined fructose + glucose (2% below > 60 g/100 g, range 57.3–76.0, median: 69.4). Although no formal industry standard exists for pH, values (range: 3.75–5.21, median: 4.1) were consistent with previous Australian (Ayton and Groves 2023) and international (Kivrak et al. 2017) studies.

### 3.3 Floral Source Diversity Was Associated With Enhanced Antimicrobial Activity

**Figure 4:**
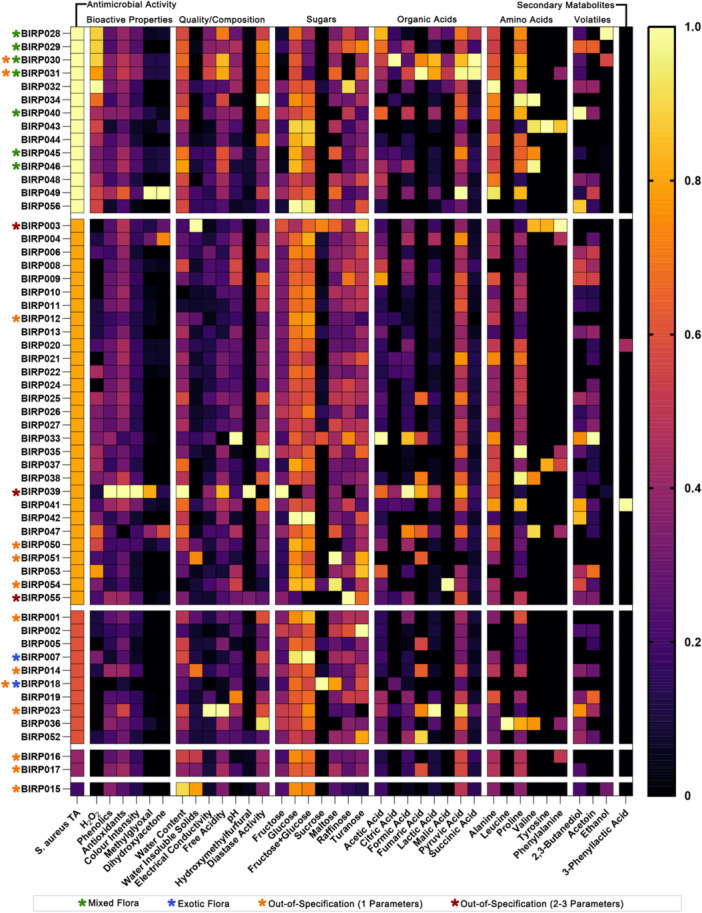
Heatmap displaying the relative levels of all measured parameters across samples. All values were normalized to a 0–1 scale, with darker colors indicating lower relative values and lighter colors indicating higher relative values. Samples are ordered from most to least active based on their *S. aureus* TA minimum inhibitory concentrations (MICs), shown in the leftmost column. Green asterisks indicate mixed flora honeys and blue asterisks indicate single origin exotic flora honeys, while orange asterisks indicate honeys with readings outside of industry standards for one parameter, and red asterisks indicate honeys with readings outside of industry standards for two to three parameters.

To visualize the multivariate structure of the dataset, a heatmap was generated of all measured parameters across the 56 honeys (Figure 4). Values were normalized to a 0–1 scale, with darker colors indicating lower relative values and lighter colors indicating higher relative values. Samples are ordered by their *S. aureus* total activity MIC values (leftmost column), providing a gradient from most to least active. Substantial heterogeneity was evident across the dataset, both within and between classes of traits. Bioactive properties such as H_2_O_2_, phenolic content, antioxidant activity, and methylglyoxal content varied markedly between samples, with no single parameter uniformly tracking with antimicrobial activity. Quality and compositional parameters were generally less variable, although occasional outliers were present. Sugars (fructose, glucose, sucrose, and minor components such as turanose and isomaltose) showed broadly consistent patterns across honeys, reflecting their central role in honey composition, while organic acids, amino acids, volatiles, and secondary metabolites showed more heterogeneous profiles, with highly active honeys tending to display elevated levels across several of these chemical categories.

All seven mixed‐flora honey samples (indicated by green asterisks) clustered predominantly in the upper portion of the heatmap, representing the most active samples. These samples generally exhibited higher H_2_O_2_ production, but their enhanced bioactivity was characterized by concurrent elevation of multiple chemical parameters rather than dominance by a single compound. Consistent with this, methylglyoxal and its precursor dihydroxyacetone were either absent or present only at negligible concentrations (≤ 44 mg/kg MGO; ≤ 149 mg/kg DHA), indicating that non‐peroxide activity was not attributable to MGO. In contrast, the two single‐origin exotic floral honeys (indicated by blue asterisks) clustered in the lower third of the spectrum, showing reduced values across bioactive categories and correspondingly weaker antibacterial activity. These patterns suggest that enhanced honey bioactivity reflects diverse, multicomponent chemical profiles, with no single driver universally responsible. Honeys with one or more quality and compositional parameters outside industry standards (indicated by orange and red asterisks) were dispersed throughout the heatmap, showing no consistent association with activity.

### 3.4 Multiple Chemical Factors Contributed to Antimicrobial Potency

**Figure 5:**
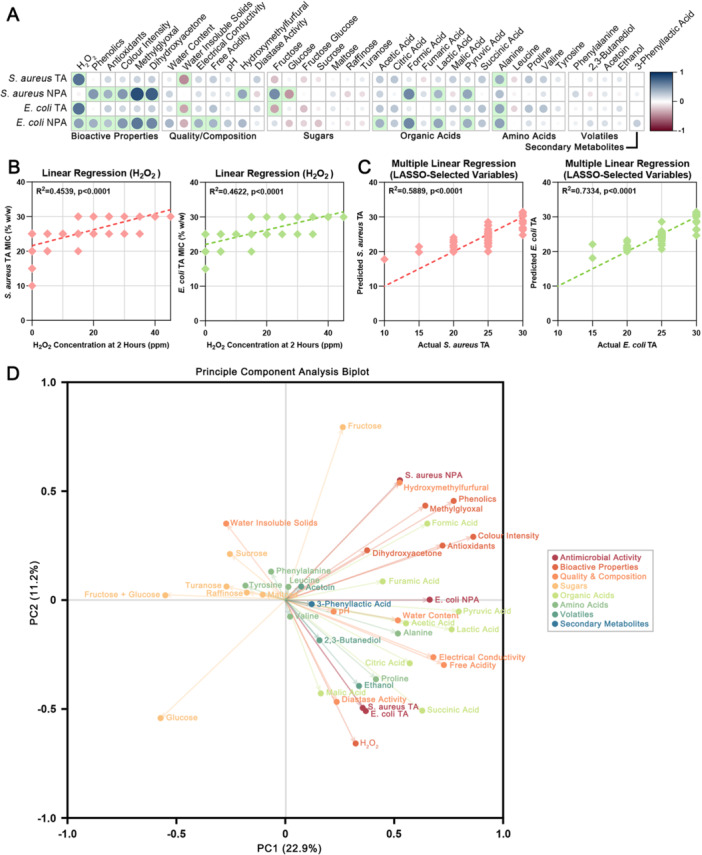
Investigating the drivers of antimicrobial activity in honey. MIC values have been reversed so that positive correlations indicate properties that align with increased antimicrobial activity. (A) Correlation matrix showing the strength and direction of associations between antimicrobial activity (horizontal) and physical and chemical properties (vertical). The size and color of the circle corresponds to the strength of the association with statistically significant correlations (*p* < 0.05) shaded in green. (B) Linear regressions showing that H_2_O_2_ alone is significantly correlated with total activity against both *S. aureus* (left; *R* ^2^ = 0.4539) and *E. coli* (right; *R* ^2^ = 0.4622). (C) Multiple linear regression using LASSO‐selected variables improves predictive power for both *S. aureus* (left; *R* ^2^ = 0.5889; variables: H_2_O_2_, antioxidants, water insoluble solids, alanine, and valine) and *E. coli* (right; *R* ^2^ = 0.7334; variables: H_2_O_2_, antioxidants, dihydroxyacetone, water insoluble solids, fructose, sucrose, maltose, turanose, acetic acid, fumaric acid, alanine, leucine, valine, acetoin). (D) Principal component analysis biplot reveals the multivariate complexity of honey.

To investigate the drivers of antimicrobial activity, associations between measured parameters and honey bioactivity were examined. As noted previously, a small number of honeys (BIRP003, BIRP039, BIRP055) had multiple quality and compositional parameters outside industry standards. To assess the impact of these outliers, predictive analyses were conducted both including and excluding these samples. Results were qualitatively consistent, indicating that these outlier samples did not affect the overall patterns. Pairwise correlations were calculated using Spearman's rank correlation, which does not assume linear relationships between variables (Figure 5A). Several bioactive properties correlated significantly with total activity, with H_2_O_2_ showing the strongest associations with activity against both pathogens. Additional moderate (0.4 ≤ *R* ^2^ ≤ 0.7) to strong (> 0.7) correlations were observed between antimicrobial activity and phenolic content, antioxidant activity, color intensity, and methylglyoxal content.

Linear regression analysis, which applies stricter assumptions of linearity, confirmed that H_2_O_2_ alone was the most predictive single variable for total antimicrobial activity (Figure 5B). H_2_O_2_ explained 45.4% of the variation in *S. aureus* total activity (*R*² = 0.4539, *p* < 0.0001) and 46.2% of the variation in *E. coli* total activity (*R*² = 0.4622, *p* < 0.0001). The strong linear relationships demonstrated consistent dose‐dependent effects, with higher H_2_O_2_ concentrations corresponding to increased antimicrobial potency for both bacterial pathogens.

Multivariate modeling using least absolute shrinkage and selection operator (LASSO) regularization substantially improved predictive power (Figure 5C). LASSO is a machine learning technique that automatically selects the most informative variables while preventing overfitting by penalizing model complexity. The LASSO‐optimized multiple linear regression models explained substantially more variation than H_2_O_2_ alone, explaining 58.9% of the variation in *S. aureus* total activity (*R*² = 0.5889, *p* < 0.0001) and 73.3% of the variation in *E. coli* total activity (*R*² = 0.7334, *p* < 0.0001). For *S. aureus*, the retained predictors were H_2_O_2_, antioxidants, water‐insoluble solids, alanine, and valine, while for *E. coli* they were H_2_O_2_, antioxidants, dihydroxyacetone, water‐insoluble solids, fructose, sucrose, maltose, turanose, acetic acid, fumaric acid, alanine, leucine, valine, and acetoin. Notably, the variables retained differed between the two models and were not always those most strongly correlated in the univariate analysis, indicating that while multivariate models can capture bioactivity more effectively, they represent data‐driven fits rather than universally predictive tools.

Principal component analysis of the complete chemical dataset further underscored the multivariate complexity underlying honey composition and bioactivity (Figure 5D). Bioactive traits such as H_2_O_2_, phenolics, antioxidants, and methylglyoxal clustered with total activity measures, indicating that these properties tend to co‐vary with antimicrobial potency. In contrast, the dominant sugars separated strongly along PC1, largely independent of antimicrobial traits, reflecting their role as core nutritional rather than bioactive components. Several organic acids, amino acids, and volatiles dispersed across both axes, showing that their contributions to activity are more variable and context dependent. The two bacterial species were tightly clustered for total activity but not for non‐peroxide activity, highlighting that while overall antibacterial strength shares common chemical drivers, species‐specific differences emerge once peroxide contributions are removed. Collectively, these analyses demonstrate that honey bioactivity emerges from complex chemical interactions, with the relative influence of individual compounds differing between samples rather than reflecting a universal set of biomarkers.

## 4 Discussion

Amid the growing global challenge of antimicrobial resistance, natural products such as honey are receiving renewed attention for their broad‐spectrum activity and low likelihood of inducing bacterial resistance. This comprehensive analysis of New South Wales honeys provides critical insights into the antimicrobial potential of Australian native flora‐derived honeys at a pivotal moment for the industry. Against the backdrop of devastating 2020 bushfires, which disrupted ecological networks and threatened beekeeping livelihoods, our systematic evaluation reveals the remarkable therapeutic potential of honeys from Australia's native flora and provides new directions for sustainable industry recovery.

A particularly striking finding was the superior performance of mixed‐flora honey samples, which clustered among the most active in our dataset (Figure 4). Floral source determination for these samples was made by professional beekeepers, which is standard practice in the Australian beekeeping industry and considered more reliable than melissopalynological (pollen) analysis, as bees may forage on eucalypt nectar while collecting pollen from different understory species to meet nutritional needs (Islam et al. 2022; Sniderman et al. 2018). In botanically diverse Australian landscapes, this decoupling of nectar and pollen sources is well documented and can lead pollen‐based methods to misrepresent the true nectar origin of honey. Honey is classified as “mixed flora” when bees forage across diverse plant communities without a single dominant nectar source. The mixed‐flora designation in our study is supported by the greater chemical complexity observed in these samples (Figure 4), consistent with contributions from multiple nectar sources rather than a single floral origin.

The high bioactivity of the seven mixed‐flora samples challenges conventional industry assumptions where monofloral honeys often command premium prices and are presumed to offer superior bioactivity. This enhanced potency may reflect additive or synergistic effects among diverse bioactive compounds supplied by multiple plant species, highlighting an important direction for future mechanistic and chemical analyses. Such chemical richness may also signal the indirect influence of bee health, as access to diverse and nutritionally balanced forage supports colony vitality and gland function—factors known to affect enzyme activity and thus honey chemistry (Di Pasquale et al. 2013; Wright et al. 2018). Australia's complex ecosystems, where species like *Eucalyptus*, *Leptospermum*, and *Melaleuca* often co‐occur with numerous understory plants, create opportunities for both chemical and nutritional diversity unavailable in more homogeneous or managed landscapes (Harden 2000).

In contrast, our two single‐origin exotic floral honeys (BIRP007 and BIRP018, produced from introduced European plants) showed notably reduced bioactivity across multiple parameters, although the small sample size precludes strong generalizations. Previous work supports the idea that exotic honeys may have reduced antibacterial activity, though this is species‐dependent. In their 2011 survey, Irish et al. found that two of three clover honeys had relatively high activity but the third was inactive, while three of three coriander honeys were inactive (Irish et al. 2011). This pattern aligns with hypotheses that co‐evolutionary relationships between native Australian plants and local environmental stressors have selected for enhanced production of defensive secondary metabolites, which are subsequently concentrated in honey through nectar collection (Isah 2019; Nicolson 2022). Collectively, these findings emphasize the value of maintaining floral diversity and prioritizing native forage to support honey bioactivity and colony health, rather than relying on monoculture or non‐native plant sources.

The antimicrobial potency of New South Wales honeys is evident in a global context. Across the 56 samples tested, 77% achieved MIC values of 10% (w/w) or lower against both *S. aureus* and *E. coli*, with 25% achieving MIC values of 5% (w/w) or lower against at least one pathogen (Figure 2A). This level of activity positions Australian honeys favorably within the global landscape, where many non‐manuka varieties require concentrations of ~3%–30% (w/w) for comparable inhibition (Grecka et al. 2019; Zainol et al. 2013; Jantakee and Tragoolpua 2015; Salonen et al. 2017; Ronsisvalle et al. 2019). While medicinal manuka honey remains the benchmark with MIC values often below 5% for bacterial pathogens (Albaridi 2019), many Australian native flora honeys matched or approached this potency. Mechanistically, where manuka honey activity is largely attributed to methylglyoxal (Cokcetin et al. 2016), the Australian native flora honeys employ multiple pathways including H_2_O_2_ and plant‐derived phenolics. These distinct pathways may be suited to different clinical or therapeutic applications, for example, phenolic‐rich honeys may be more suited for chronic wounds that require sustained anti‐inflammatory effects (Zhao et al. 2016). Unlike manuka honey, which is produced from a small range of *Leptospermum* species and is therefore limited in volume, Australia's extensive and diverse native forests could support scalable production of high‑bioactivity honeys.

Most quality parameters were within accepted industry standards and were similar to results from other Australian and international studies (Ayton and Groves 2023; Kivrak et al. 2017; Boussaid et al. 2018; Chakir et al. 2016; Kahraman et al. 2010; Laaroussi et al. 2020). Thresholds were most commonly exceeded for electrical conductivity and water‐insoluble solids, though this likely reflects the use of raw, unfiltered samples obtained directly from beekeepers. A small number of atypical samples exhibited elevated hydroxymethylfurfural, reduced diastase activity, and unusual sugar ratios, indicative of prolonged or suboptimal storage. These results highlight both the natural variability of New South Wales honeys and the influence of environmental conditions and handling practices on composition. Maintaining proper storage conditions is particularly critical for preserving bioactive potential, as our previous research demonstrated that antimicrobial activity can be retained for 15–17 years when under optimal storage conditions (Fernandes et al. 2024a).

To understand how chemical complexity translates into antimicrobial efficacy, the relationships between individual compounds and bioactivity were examined. Our analyses represent the most comprehensive combined antimicrobial and chemical profiling of Australian honeys to date. Mechanistic insights from our modeling reinforce that honey antimicrobial activity emerges from complex chemical interactions rather than single dominant compounds (Figure 5A–D). While H_2_O_2_ explained 45%‐46% of variation in total antimicrobial activity, LASSO regression models incorporating multiple chemical parameters achieved substantially improved predictive power (59% for *S. aureus*, 73% for *E. coli*). This aligns with international studies on floral honeys, which similarly demonstrate that H_2_O_2_ is a major but not the singular contributor to antibacterial activity (Bucekova et al. 2019; Farkasovska et al. 2019). The specific variables retained differed between bacterial species, indicating pathogen‐specific mechanisms of action that are likely due to underlying differences in bacterial physiology such as cell wall structure, membrane permeability, and susceptibility to oxidative stress.

The amino acid signatures in the different honey samples most likely reflect underlying nutritional and physiological processes within the colony rather than any direct effect on antimicrobial activity (Pasupuleti et al. 2017). The identification of alanine, valine, and leucine as significant predictors of activity may indicate the presence of good forage and healthy colonies, as pollen from native Australian flora varies widely in crude protein and amino acid composition (Somerville and Nicol 2006; Rayner and Langridge 1985). While some native pollens are nutritionally adequate, pollen from other taxa (particularly *Eucalyptus* spp.) is frequently deficient in one or more essential amino acids and falls below the thresholds required for optimal bee development (Somerville and Nicol 2006; Rayner and Langridge 1985). This nutritional heterogeneity could explain why colonies foraging on diverse floral assemblages perform better than those restricted to single sources. Behavioral studies also show that bees actively compensate for such imbalances by selecting complementary pollen mixtures when available, suggesting an adaptive strategy to achieve a balanced amino acid intake (Hendriksma and Shafir 2016). Consequently, landscapes with high botanical diversity provide a nutritional buffer that supports colony resilience and productivity, indirectly contributing to the chemical richness and antimicrobial potency of their honeys (Zhao et al. 2016). Our recent studies demonstrate that colony health directly influences hive products, with healthy colonies producing significantly more bioactive honey and pollen (Fernandes et al. 2023, 2024b).

These mechanistic insights, combined with consistently high activity, reveal substantial opportunities to develop Australian honey as a therapeutic alternative. The breadth of antimicrobial activity across diverse Australian honeys suggests untapped potential for clinical and commercial development. Given the growing burden of pharmaceutical waste (Wilkinson et al. 2022) and the non‐toxic nature of medicinal honey (Fernandes et al. 2024a), Australian honeys could represent both an environmentally sustainable and therapeutically effective alternative to conventional antimicrobials for topical wound management, including chronic wounds, burns, and surgical site infections. As global demand grows for natural antimicrobials, Australian honeys could represent a resilient and diversified supply chain.

To realize the therapeutic potential of Australian honeys, sustainable beekeeping practices that maintain floral diversity are essential: our findings on the superior performance of mixed‐flora honeys suggest that intensive monoculture approaches, while potentially increasing consistency, may compromise the bioactive potential that makes honey valuable for therapeutic applications. This also supports bee health, as access to diverse floral resources is known to improve honey bee colony health and resilience (Di Pasquale et al. 2013; Fernandes et al. 2022; Brodschneider and Crailsheim 2010), likely creating a positive feedback loop where ecosystem diversity supports colony health, which in turn produces higher‐quality therapeutically valuable products.

## 5 Conclusion

Our findings highlight the potential premium market value of ecologically diverse honey products and underscore the importance of conserving Australia's native ecosystems. The antimicrobial and chemical profiling approaches demonstrated here provide a framework for quality assessment and standardization, which may support future regulatory consideration and the informed development of Australian honeys for therapeutic use. As the Australian honey industry rebuilds from recent bushfire impacts, these insights suggest that prioritizing ecosystem diversity over monocultural production systems could unlock both enhanced bioactivity and greater resilience to future environmental disturbances.

## Author Contributions

**Kenya E. Fernandes:** conceptualization, investigation, writing – original draft, methodology, validation, visualization, writing – review and editing, formal analysis, data curation, supervision. **Andrew Dong:** investigation, methodology. **Jamie Ayton:** conceptualization, investigation, methodology, writing – review and editing, formal analysis. **Leanne Groves:** investigation, methodology. Kerrie Graham: investigation, methodology. Peter Brooks: investigation, methodology. **Nural Cokcetin:** conceptualization, project administration, writing – review and editing. **Dee A. Carter:** conceptualization, project administration, writing – review and editing, supervision.

## Ethics Statement

The authors have nothing to report.

## Conflicts of Interest

The authors declare no conflicts of interest.

## Supporting information


**Supporting Table S1:** All activity, physical, and chemical property data.

## Data Availability

The data that supports the findings of this study are available in the supporting material of this article.
